# Beyond boundaries a hybrid cellular potts and particle swarm optimization model for energy and latency optimization in edge computing

**DOI:** 10.1038/s41598-025-90348-x

**Published:** 2025-02-20

**Authors:** Dinesh Sahu, Nidhi, Shiv Prakash, Priyanshu Sinha, Tiansheng Yang, Rajkumar Singh Rathore, Lu Wang

**Affiliations:** 1 SCSET, Bennett University, Plot Nos 8, 11, TechZone 2, Greater Noida, Uttar Pradesh 201310 India; 2 Department of Electronics and Communication, University of Allahabad, Prayag Raj, Uttar Pradesh India; 3 Department of Electronics and Communication, University of Allahabad, Prayag Raj, Uttar Pradesh India; 4 University of South Wales Pontypridd, Pontypridd, UK; 5 Cardiff School of Technologies, Cardiff Metropolitan University, Cardiff, UK; 6 Xi’an Jiaotong-Liverpool University Suzhou, Suzhou, China

**Keywords:** Edge computing, Cellular Potts model, Particle swarm optimization, QoS optimization, Resource scheduling, Energy efficiency, Latency reduction, Computer science, Information technology

## Abstract

The need to compute data in real-time and manage resources in environments with distributed computing has given edge computing significant importance. However, one of the most critical tasks regarding resources has been to schedule and optimize them in accordance with energy consumption and delay time. These challenges has been addressed in this paper with the introduction of a new integrated method that assumes the Cellular Potts Model and Particle Swarm Optimization. The Cellular Potts Model is used to capture local interaction and dependencies of resources, while PSO acts as a global optimizer for scheduling reducing latency and energy consumption. Based on these considerations, the primary research goal of this work is to mitigate the QoS requirements like energy consumption and end-to-end delay using CPM—spatial modeling complemented by PSO - the global optimization. Based on experimental analysis, the authors of the paper argue that the newly proposed Hybrid model consumes less energy and has less processing time than Round-Robin, Random Offloading, and Threshold-Based techniques. In addition, the approach achieves higher scalability and can perform a large of tasks and edge nodes with a high QoS while working in a resource-limited environment. This paper contributes to presenting the integration procedure of the CPM’s local optimization with the PSO’s global search, which offers high-performance and real-time solutions for resource scheduling in the edge computing environment. The results presented in the paper show that the proposed hybrid CPM-PSO model can offer greater potential as a tool for energy-constrained and time-sensitive applications within the future development of edge computing.

## Introduction

The rapid growth of latency-sensitive, data-intensive applications combined with the rapid proliferation of Internet of Things (IoT) devices has brought back edge computing into the limelight^1,2^. The new model, in which computing resources are brought close to end users and devices, is different from the traditional cloud computing model, whereby data must roam long distances from the edge of the network to large centralized data centers. This architectural shift reduces network latency, gives bandwidth saving, and brings real-time analytics capabilities leading to a number of applications such as connected autonomous vehicles (CAVs), augmented reality (AR), and smart cities^3,4^.

Nevertheless, this implies other challenges for deploying computational resources to the network edge. The most important among them is to allocate and schedule tasks efficiently across heterogeneous resource-constrained edge nodes^5^. With increasingly sophisticated applications, the trade-offs in meeting these conflicting objectives of reducing energy consumption and keeping latency low grow more and more difficult. Traditional resource scheduling strategies, such as Round-Robin or Threshold Based, fail to reactively provide adaptive scheduling under changeable workload patterns, network conditions, and resource states^6^. This, therefore, demands intelligent and adaptive resource scheduling techniques that can deal with the inherent complexity as well as the uncertainty of the edge environments.

### Problem statement

Many existing scheduling algorithms tend to apply simplistic heuristics or static rules when making scheduling decisions, resulting in sub-optimal energy efficiency and reduced latency^7^. However, these algorithms do not exploit the spatial and temporal dynamics of the distribution of the resource across the supply chain and do not adequately explore the vast solution space for defining the schedule. Furthermore, most existing strategies adopt a single optimization technique, restricting them to under-performing in environments with complicated and changing dynamics^8^. Thus, unnecessary energy overhead, reduced operational lifetime of devices, and increased latency caused by the edge computing system hold back its performance as well as the performance of latency-critical applications.

For example, a pervasive edge environment with several resource nodes (edge servers) that perform several different tasks under very different computational burdens. Static scheduling techniques may result in inefficient utilization of resources and then can cause some nodes to become overloaded, increasing both their energy usage and processing delays. On the other hand, a more adaptive approach based on robust modeling and search techniques can reconfigure a dynamic allocation strategy to percolate energy consumption while satisfying latency requirements.

### Contributions

**Fig. 1:**
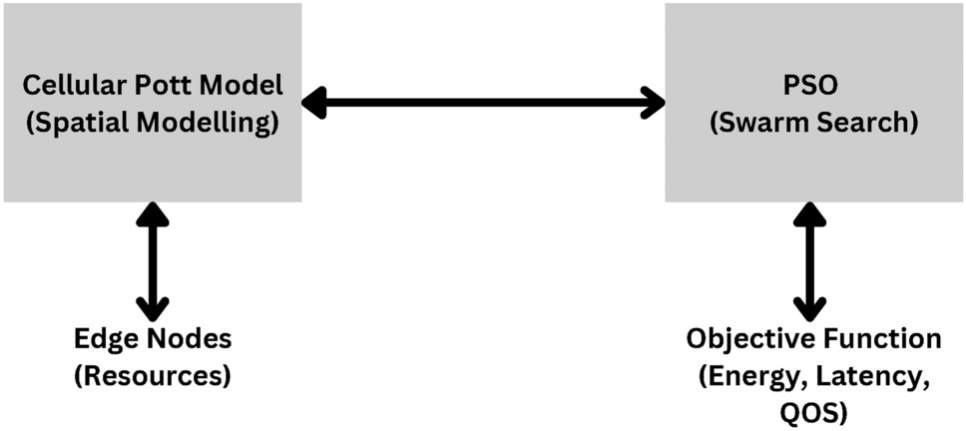
Conceptual architecture of the hybrid CPM-PSO approach for resource scheduling.

In contrast to other strategies for scheduling, the proposed one uses the Cellular Potts Model as a basis for spatially-conscious scheduling and complements it with Particle Swarm Optimization to obtain an optimum solution. This simplifies the process by minimizing the overall search space drastically and optimizes the convergence and efficiency of the resources. This paper presents a novel hybrid approach using the Cellular Potts Model (CPM), along with Particle Swarm Optimization (PSO), to improve end-user QoS in terms of both energy and latency. The QoS parameters like energy efficiency and latency are significant quality measures in edge computing. Although these parameters are not new, the question of how they can be optimized remains a problem in dynamic and resource-scarce conditions. This research advances this knowledge area by using a newly developed Cellular Potts Model-Particle Swarm Optimization approach to address these objectives more efficiently than conventional methods. This work proposes a new approach to the Cellular Potts Model (CPM) in conjunction with Particle Swarm Optimization (PSO) to discuss the two objectives of energy consumption and low latency in edge computing. While CPM affords spatial modeling means to cater for interaction and dependency of edge nodes at a more local level; PSO provides edge resources with a strong global optimization technique for scheduling. The hybrid approach just combines the ideas of two methods, which will produce a feedback loop that constantly improves the spatial configuration and the scheduling decision; and provides a new perspective that is not discussed in the current literature. In edge, fog, and cloud computing, resource scalability and adaptability are still major problems due to the workload variability and changing resource environment. To overcome these challenges, this work develops a CPM-PSO method that can facilitate improved adaptability to the variation in workload and efficient resource scheduling as the number of tasks and edge nodes grows. To allow more realistic and constraint-aware scheduling decisions, the CPM component provides a spatial modeling framework that simulates the interactions and configurations among resource nodes^9^. CPM allows modeling both resource states and their spatial relations with neighborhoods where the CPM is capable of capturing complex spatial dynamics that are ignored in more traditional methods of analysis. However, a well-known metaheuristic algorithm, PSO inspired by the flocking behavior of bird flocks, efficiently explores the solution space to discover near-optimal scheduling configurations^10^. It uses swarm intelligence and probabilistic rules to guide the search process using initial CPM-based states for state improvement and overall performance improvement. Figure 1 Hybrid CPM-PSO conceptual architecture for resource scheduling in edge computing environments. The CPM layer models resource configurations and spatial relations and searches for near-optimal scheduling solutions, with PSO refining the CPM to obtain such solutions.The reason why the Hybrid CPM-PSO approach was proposed is due to the fact that in edge computing systems there is a need to distribute the resource allocations locally, while at the same time ensuring a Global Optimization. Round-robin and Threshold-Based forced policies, do not perform sensible changes in response to changing workload conditions while, metaheuristic-based methods including PSO and GA can converge to a non-optimal solution very quickly while the optimization process is highly time-consuming in distributed systems. The CPM is a good match to a spatially aware scheduling paradigm since it operates on ‘local’ interactions and resource constraints between the edge nodes. On the other hand Particle Swarm Optimization (PSO) improves the global optimization by optimizing the configuration estimated using CPM. However, when these two techniques are integrated, the CPM-PSO algorithm has the advantage of less energy consumption, less delay, and better scalability as compared to the other scheduling algorithms. It also reduces the computational complexity of metaheuristics because PSO is initiated with semi-defined CPM topologies, shortening dynamism and enhancing resource optimization. Key contributions of this paper are as follows:

1. **Novel Hybrid Methodology:** In order to balance spatial modeling with global optimization benefits, we introduce a CPM-PSO hybrid framework to optimize resource scheduling.
2. **QoS Improvements:** The considered approach aims at minimizing energy consumption and latency both of which are considered critical QoS parameters in edge computing.
3. **Performance Validation:** To demonstrate the efficacy of the hybrid CPM-PSO method, the approach is compared with classical methods (Round Robin, Random Offloading, Threshold Based), as well as well-known heuristics (PSO alone, Genetic Algorithm), and we show that it achieves significant improvements in both energy efficiency and latency reduction.
4. **Scalability and Adaptability:** We also show that the model scales well with the number of tasks and edge nodes, and is robust to dynamic changes in the environment.

### Structure of the paper

The remainder of this paper is organized as follows: In section “Related work”, we present a comprehensive review of related work, emphasizing existing resource scheduling strategies in edge computing and discuss why they fall short. In section “Proposed methodology”, the proposed hybrid CPM-PSO framework is described, and the system model in addition to the underlying mathematical formulations of various components has been presented. In section “Environmental setup and parameters” we propose the experimental setup, metrics, as well as scenarios with which we evaluate the proposed approach. In section “Results and discussion”, we present and discuss our experimental results with comparisons to baseline algorithms and limitations of the proposed method. Section “Conclusion and future scope” finally concludes the paper with a summary of key findings and further research directions.

## Related work

Traditional resource scheduling algorithms in edge computing environments usually use fixed and heuristic rules that are inadequate for elastic workloads and dynamic network conditions. For example, Round-Robin (RR) scheduling schedules tasks evenly over all runnable available nodes without taking into account the inherent heterogeneity of available resource sizes to available nodes, which leads to possible under-utilization or load-balancing issues^11,12^. Due to Random Offloading (RO)^13^, which randomly assigns incoming tasks to edge nodes, the resulting latency and corresponding energy consumption are unpredictable and ineffective. In parallel, threshold-based (TB) methods depend on predefined thresholds (such as values of CPU utilization) to decide to offload, but they usually do not address the complexities in the dynamics of real-time systems^14,15^.

These classical methods are simple, fast, and easy to implement, but their sensitivity to the machining environment, limited scalability, and inability to accommodate unexpected changes have made them inadequate for the nature of the new manufacturing environment^16,17^. However, studies have shown that these approaches cannot respond quickly to rapidly varying workloads within an Edge computing environment, thus causing either a large delay for some tasks, or massive energy consumption at some nodes^18^. As edge computing ecosystems grow in scale and complexity, conventional answers to the same problem such as RR, RO, and TB do not scale effectively^19,20^. To improve the QoS parameters like lower latency and less energy consumed, this shortfall has driven researchers towards more intelligent and flexible solutions.

Due to the limitations of traditional methodologies, metaheuristic algorithms have been pursued to solve the problem of resource scheduling and optimization in edge environments. Two widely studied techniques using evolutionary or swarm intelligence concepts to search for near-optimal configurations are Particle Swarm Optimization (PSO)^21,22^ and Genetic Algorithms (GA)^23–25^. Such methods are capable of working with large, intricate search spaces and adapting their pattern of search accordingly over time based on objective function evaluations.

For instance, PSO has been used to assign dynamics tasks to edge nodes to reduce latency and energy consumption^26,27^. In addition, GA-based approaches have been exploited to minimize operating costs under load balancing constraints^28,29^ by iteratively evolving a population of candidate solutions. Furthermore, other metaheuristics, including Ant Colony Optimization (ACO)^30^, Simulated Annealing^31^ and Tabu Search^32^, have been used to tackle the scheduling of resources. While their reported performance suggests moderate success, these metaheuristics typically work without an explicit spatial representation and just use abstract solution representations that may not represent the spatial constraints and edge interactions well^33^.

To simulate morphological changes in tissues, originally, the Cellular Potts Model (CPM) was introduced in the context of biological cell modeling^34^. In particular, CPM has been used for a variety of computational modeling settings such as material science, image segmentation, and pattern formation studies^35^ since it can represent and manipulate spatial configurations. The CPM uses a lattice of cells to treat resources, permitting the inclusion of spatial relationships as well as local interaction rules; this provides a more realistic representation of how spatial relationships and workload reconfiguration of edge nodes over time occur^36^. Resource management and power quality have been greatly enhanced in recently developed optimization techniques for distributed systems. The energy efficiency of meta-heuristic methods like flower pollination, honey badger optimization, and a hybrid Jaya-grey wolf optimization has also been effectively used to reduce harmonic distortion and stabilize system performance under variable conditions^37–40^. Later, the complex challenges were addressed by innovative integrations of neural networks and optimization algorithms which overcame typical limitations^41^. These studies serve as a basis for the proposed hybrid CPM-PSO method and extend these improvements to optimize energy efficiency, latency, and scalability in edge computing domains.

When moving to resource scheduling, CPM can be used to map disjunct heterogeneous resources and their states to spatial configurations, serving as a framework for simulating, evaluating, and comparing different allocation patterns subject to different constraints. Although CPM has the potential, it alone does not do a global search for the best solutions. It does not do this; instead, it works superbly on energy minimization and locally adapting configurations through neighborhood interaction. The combination of CPM and a global optimization technique can better guide the search process with configurations that minimize latency and energy usage.

### Gap analysis

Traditional methods are not able to adaptively schedule resources to achieve QoS goals, while PSO and GA both provide global optimization ability but are insufficient in native spatial modeling. On the other hand, CPM gives a spatially rich representation with no robust search mechanism. This observation highlights a critical gap: integration of a spatial modeling method (like CPM) with a global optimization metaheuristic (like PSO).

While this gap cannot be bridged by either CPM or PSO alone, we can bridge it by hybridizing them. Providing a realistic spatial landscape for the CPM in resource scheduling, it captures how tasks and resources interact at the local level. PSO can then navigate this landscape globally to find low-latency/low-energy configurations. Existing resource scheduling methods tend to compromise between localized optimization and global task allocation. By integrating CPM spatial modeling with PSO global search in this work, adaptability, and performance are better supported in dynamic edge computing environments. The synergy that this approach offers is composed of a more robust, adaptive, and scalable solution than any of the approaches could achieve individually. To mitigate the identified problems, this paper proposes a new hybrid CPM-PSO framework that can potentially exceed traditional and meta-heuristics alone approaches in producing energy-efficient and low-latency resource scheduling.

## Proposed methodology

### Overview of the proposed hybrid model

We present a hybrid model that consists of two key components: a Cellular Potts Model (CPM), a particle modeling formalism, and Particle Swarm Optimization (PSO), an optimization technique, to model and optimize resource scheduling for edge computing based on demand prediction. CPM (Fig. 1) represents resources in a spatially aware fashion, capturing the evolution of task allocation patterns through localized interactions and constraints^36^. By contrast, PSO is applied as a global optimization method to explore and refine the scheduling configurations with respect to key Quality of Service (QoS) objectives such as energy consumption and latency^42^. Energy efficiency and latency are QoS parameters that have a major influence on the applicability and effectiveness of using edge computing systems. These metrics were chosen as the subject of this paper because they are relevant for assessing the effectiveness of resource scheduling frameworks. The novelty of this research is based on the integrated CPM-PSO methodology used to improve these conventional parameters whereby the hybrid model employs spatial and global resource allocation. As it can be concluded with reference to the given findings, this methodology outperforms the existing techniques in attaining enhanced improvements in these pivotal criteria. By integrating CPM and PSO:

1. Using local “energy” interactions and constraints, CPM models dynamic and spatial distribution of tasks over edge nodes, and moves towards feasible scheduling configurations.
2. The globally optimal or near-optimal solutions to search in the generated CPM configuration space are candidate globally optimal or near-optimal resource scheduling allocations that minimize a multi-objective cost function (for example weighted sum of energy and latency).

This hybrid synergy addresses the limitations of using CPM or PSO alone: The spatial modeling in CPM does not inherently have robust global search, and PSO can leverage a spatially enriched formulation that prevents it from converging to suboptimal solutions. The proposed hybrid CPM model with PSO optimization is expected to cater to scalability by creating a fusion of CPM spatial modeling and PSO global optimization. In CPM, localized interactions between the edge nodes are recorded, thereby allowing the control of an increasing number of resource nodes without considerable computing costs. This is where PSO complements this by globally optimizing scheduling and guaranteeing consistency as the system expands. Adaptability is established through the feedback loop from CPM to PSO. CPM simultaneously adapts resource configurations due to local conditions, whereas PSO finely tunes these configurations in response to workload fluctuations and resource states. This way the model evolves very well to dynamic environments and still be able to deliver a high QoS regardless of the workload and availability of resources.

### Cellular Potts model (CPM) component

**Fig. 2:**
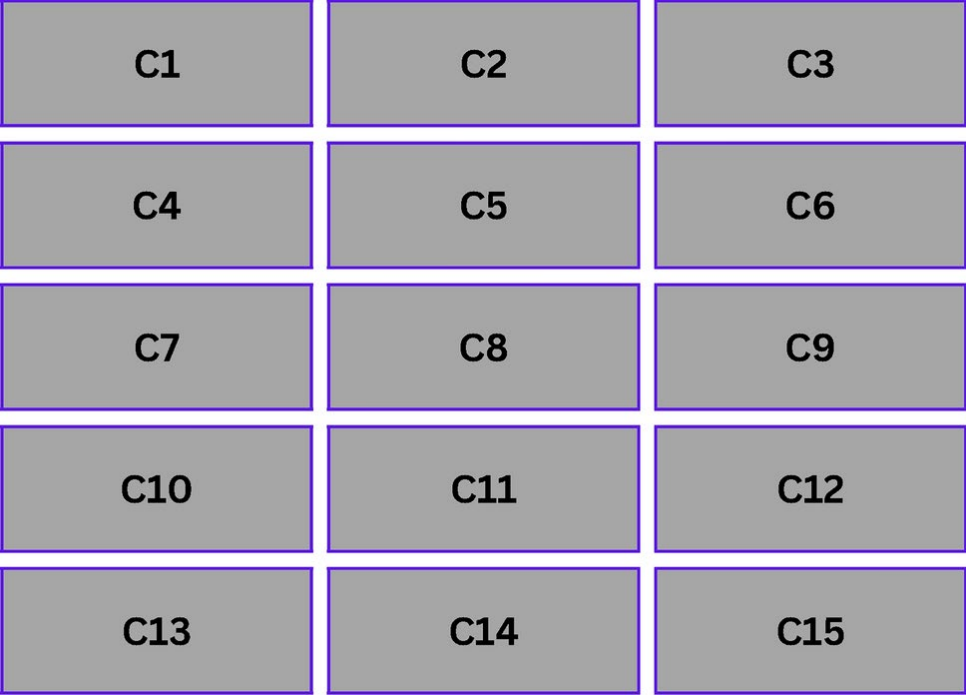
Simplified diagram of how CPM cells.

In biology, the Cellular Potts Model was first formulated to model cell sorting and morphological processes, however, it has since been used for computational modeling tasks such as resource allocation^43^. In particular, CPM is used to express a two-dimensional grid (or higher dimensional representation if necessary) of edge resources with their associated states, i.e., indicating load, capacity, and operational constraints. Each “cell” (i.e., edge node) $i$ in the CPM grid has a state $s_i$. The energy of the overall system is given by:

1 $$\begin{aligned} E = \sum _{i \in G} \left( \sum _{j \in N(i)} J(s_i, s_j) \right) + \lambda \sum _{i \in G} P(s_i) \end{aligned}$$

where $G$ is the set of grid cells (edge nodes), $N(i)$ is the neighborhood of cell $i$, $J(s_i, s_j)$ represents the interaction cost between cells $i$ and $j$, capturing load imbalance or capacity conflicts, $P(s_i)$ is a penalty function encoding constraints such as maximum CPU usage or memory limits and $\lambda$ is a weighting factor controlling the relative influence of penalty terms. Changing cell states probabilistically according to such rules minimum $E$ makes the CPM evolve. A proposed state change is accepted if it decreases energy, or with probability: $\exp \left( -\frac{\Delta E}{T}\right) .$ In summary, if the simulated annealing principle works here, any algorithm that increases energy, where T is a “temperature” parameter, decreases over time. Figure 2 gives a simplified diagram of how CPM cells might look in a 2D layout where each cell $C_i$ represents an edge node with state $S_i$

The Cellular Potts Model (CPM) is a powerful framework for modeling and optimizing complex systems owing to its key properties. An important aspect is spatial interactions in which cells represent adjacency constraints. So, resource usage in one node is important to its neighborhood nodes, albeit through network bandwidth or proximity-limited interference. They capture dependencies and limitations associated with distributed systems, and local and regional effects are incorporated into the model.The second important property is that probabilistic updates can be performed in the CPM to stochastically explore feasible configurations. Rather than deterministically picking out and settling into a possibly suboptimal local minimum, the model probabilistically updates cell states. This stochastic feature of the system enables it to escape rigid local minima and examine a more comprehensive solution space; thereby it is more robust to find near-optimal configurations. Lastly, the handling of constraints in CPM is highly adaptable as penalty functions and interaction energies are used. These elements can be precisely tuned to emphasize particular objectives (e.g. resource usage, load balancing, or conformity to other specialized constraints such as co-location policies). Because of the ability to flexibly tune the CPM based on specific applications’ needs, the CPM can support applications as diverse as grid computing and task offloading in edge and fog networks.

### Particle swarm optimization (PSO)

Particle Swarm Optimization (PSO) is a type of nature-inspired optimization technique that mimics the flocking of birds or schooling of fish. It is a population-based algorithm where each individual in the candidate solution (population) is called a particle^44^. It is done with the idea that particles move through the search space by changing their positions and velocities on their own experience of past moves and the moves of their neighboring particles. Particles track best-known positions (personal best) and are also influenced by the best-known position found by the swarm so far (global best position). The position update is governed by two components: A combined cognitive, which tries to draw the particle towards its personal best, and social, which draws it towards the global best. The solution space is usually explored because the movement equations usually include random ones. After some iterations, the swarm finds the optimal or near the optimal solutions. The major factor of its wide use is its simplicity in handling such non-linear, multi-modal functions and it also can adapted for several variants of task scheduling, resource allocation, and feature selection. Its performance depends on inertia weight, cognitive coefficient, and social coefficient which control the trade-off between exploration and exploitation^45^.

### Hybrid CPM-PSO model for QoS optimization

A robust solution can be provided by creating a hybrid model that combines the Cellular Potts Model (CPM) and Particle Swarm Optimization (PSO) to optimize QoS parameters, namely energy consumption and latency, in resource scheduling of edge computing. The benefits of the Hybrid Model is Spatial Modeling: Interaction between resource nodes is modeled effectively by CPM and proper handling of constraints, and Although CPM defines the feasible state space within which the optimal solutions are searched for by PSO, the energy efficiency and the latency of the optimal solutions obtained are considerably better than that in PSO solely relied on to locate the global optimal solutions.

**Table 1:** Symbols and their descriptions.

Symbol	Description
**CPM parameters**	
*G*	Grid of CPM cells (edge nodes)
$s_i$	State of cell (node) *i*
$s'_i$	Proposed new state for cell *i*
$\Delta E$	Change in energy: $\Delta E = E(s'_i) - E(s_i)$
$E(s_i)$	Energy function capturing interactions and constraints
$J(s_i, s_j)$	Interaction coefficient between neighboring cells *i* and *j*
$P(s_i)$	Penalty function encoding resource constraints for cell *i*
$\lambda$	Weighting factor for the penalty term in CPM
*T*	Temperature parameter controlling probabilistic acceptance in CPM
**PSO parameters**	
*M*	Number of particles in the PSO swarm
$x_j(t)$	Position (scheduling configuration) of particle *j* at iteration *t*
$v_j(t)$	Velocity of particle *j* at iteration *t*
$pBest_j$	Personal best position found by particle *j*
*gBest*	Global best position found by the swarm
$\omega$	Inertia weight balancing exploration/exploitation
$c_1, c_2$	Acceleration coefficients guiding movement towards $pBest_j$ and *gBest*
$r_1, r_2$	Random scalars $\in [0,1]$ for stochastic velocity updates
**Objective function terms**	
$\alpha , \beta$	Weights balancing energy vs. latency in the fitness function
$\text {Energy}(x)$	Total energy consumption given scheduling configuration *x*
$\text {Latency}(x)$	Total task latency under scheduling configuration *x*
*f*(*x*)	Objective function: $f(x) = \alpha \cdot \text {Energy}(x) + \beta \cdot \text {Latency}(x)$
**Miscellaneous**	
*N*	Number of CPM cells (edge nodes)
$\text {maxIter}$	Maximum iteration count for the hybrid algorithm

**Fig. 3:**
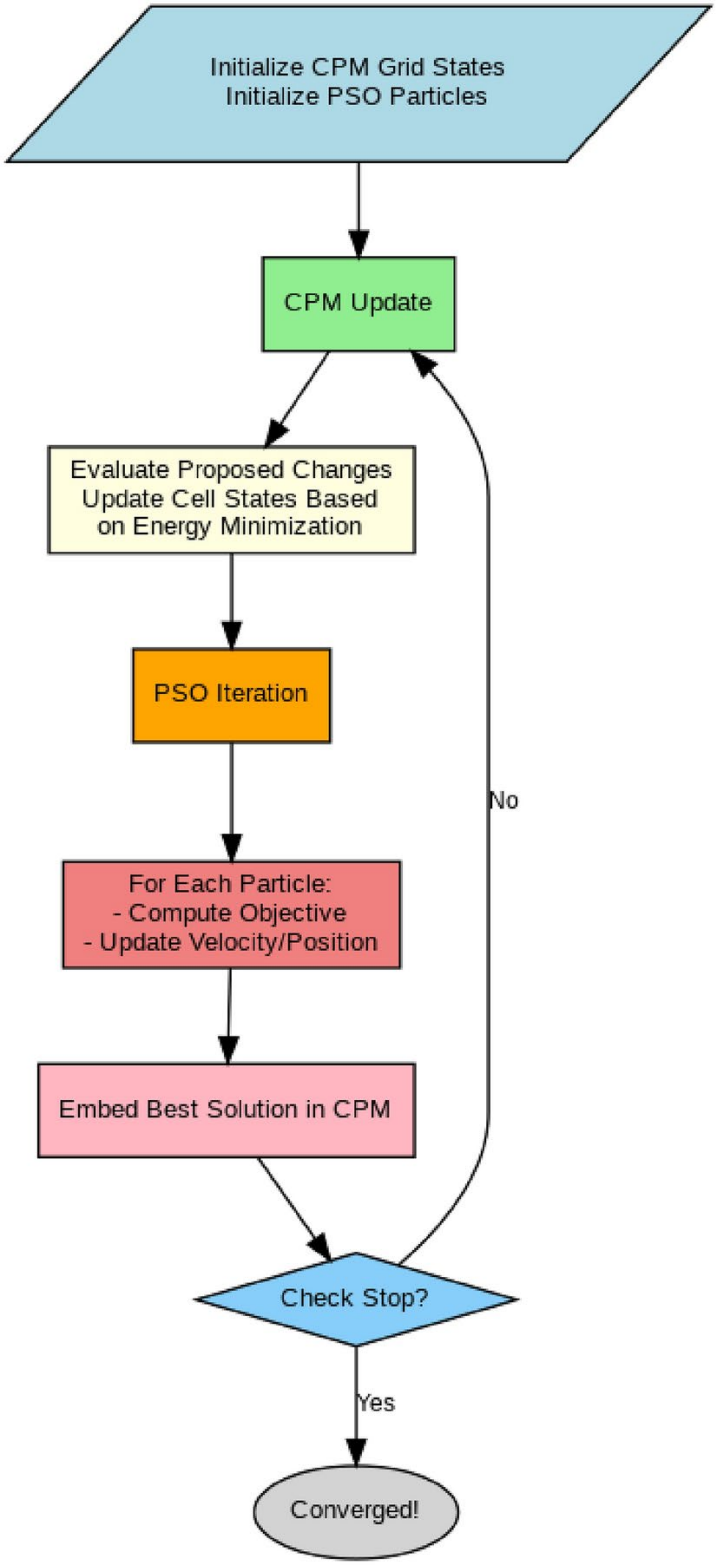
Hybrid CPM-PSO flowchart.

The core idea of the proposed hybrid optimization approach is to combine CPM and PSO in an opposing process to reach optimized scheduling. The spatial state of the edge environment is represented and evolved by the CPM, including node usage, adjacency constraints, and penalizing violations of node admissibility constraints. The PSO search space is built upon this evolving state based on each particle being a scheduling configuration that references the current CPM resource states. The best scheduling configuration discovered in each PSO iteration is fed back to CPM to influence the evolution of the state of the grid’s cell. This process will continue until convergence or when a designated termination condition has been set. The initialization process starts with the CPM grid being initialized with initial node states and initial positions of particles of PSO are randomly assigned to initial positions representing scheduling allocations. In the CPM update phase, the grid transforms along the paths that the CPM energy function determines to be locally favorable resource configurations. In the PSO optimization phase, the objective function that minimizes energy consumption and latency is calculated on each particle and the CPM state is inferred to justify its current fitness. Finally, particles update their velocities and positions towards better solutions. Finally, the feedback loop guarantees that the best solution found by PSO is ‘featured’ back to the CPM state, by integrating globally optimized scheduling decisions into the grid configuration. The steps of hybrid CPM and PSO is represented in Fig. 3. Table 1 represents a comprehensive list of all symbols and their description used in this research. Here’s a detail of how such a model could be structured:

#### Model initialization

The edge computing resources are represented as the CPM grid. The grid represents a set of resource nodes that are assigned a particular state (idle, partially loaded, or fully loaded) per cell. A probabilistic function that considers resource constraints and usage requirements is used to determine the evolving state of each cell. The particles of PSO are initialized with a swarm initialized with a swarm of particles each representing a potential solution for the resource allocation scheduling over the CPM grid. Each particle represents a position (an individual scheduling configuration) and a velocity.

#### Objective function design

The key Quality of Service (QoS) parameters that need to be optimized should be encapsulated in the objective function as the minimization of total energy consumption of the edge nodes and the task latency, consisting of both the communication and processing delays. The objective function is defined as:

2 $$\begin{aligned} \text {Objective} = w_1 \times \text {Energy}(x) + w_2 \times \text {Latency}(x) \end{aligned}$$

where $x$ is a candidate solution (particle’s position) and $w_1$ and $w_2$ are weights, which give the importance of energy and latency equivalent.

#### CPM-based state evolution

The CPM updates the resource cell’s state using a step involving interactions with neighbors and constraints on each iteration. The CPM state evolution is used to define resource allocation solutions’ feasible boundaries and constraints.

#### Particle swarm optimization (PSO)

Each particle evaluates its current position using the CPM-derived objective function and calculates the fitness value by the energy and latency performance. The particle’s velocity and position are updated using the following standard PSO equations:

3 $$\begin{aligned} v_i(t+1)= & \omega \cdot v_i(t) + c_1 \cdot r_1 \cdot (p_{\text {best}} - x_i(t)) + c_2 \cdot r_2 \cdot (g_{\text {best}} - x_i(t)) \end{aligned}$$

4 $$\begin{aligned} x_i(t+1)= & x_i(t) + v_i(t+1) \end{aligned}$$

where $p_{\text {best}}$ and $g_{\text {best}}$ are the particle’s personal best and the global best positions, respectively, $\omega$ is the inertia weight, $c_1$ and $c_2$ are acceleration constants, $r_1$ and $r_2$ are random values.

#### Hybrid optimization loop

Based on the new allocations proposed by the PSO particles, the grid is iteratively evolved in the Cellular Potts Model (CPM) to update the resource states. At the same time, each particle position is evaluated using the updated CPM grid state to refine particle positions, as well as identify the optimal solution solutions for energy efficient and low latency resource scheduling using Particle Swarm Optimization (PSO).

#### Convergence and solution selection

Such optimization process continues until it reaches a convergence criterion, e.g., reaching the predefined maximum of iterations or achieving a minimum value of threshold changes in QoS parameters. After convergence is achieved, the best solution caught during processing is then chosen as the final optimized resource scheduling configuration.

### Rationale for hybrid approach

With global optimization tasks, Particle Swarm Optimization (PSO) is ideal for adjusting the spatial configurations produced by the Cellular Potts Model (CPM) to optimize task scheduling under dynamic edge computing environments. This scalability comes for free, through its simplicity which stresses a population-based search driven by the intelligence of individual and collective agents as the workload and system sizes vary. Also, PSO’s global solution refinement complements CPM’s worldwide local optimization, and the whole resource scheduling can be done in an efficient and scalable way. Compared to other optimization algorithms, conventional PSO has been well-validated in a broad range of optimization problems and is a robust and reliable means with which to incorporate PSO into hybrid methodologies such as the proposed CPM-PSO framework.

The rationale for choosing both the Cellular Potts Model (CPM) and Particle Swarm Optimization (PSO) was based on the fact that both techniques are uniquely suited to solving resource scheduling in edge computing scenarios. CPM, a powerful framework for spatial modeling, models edge nodes as a grid of interacting cells, accurately containing localized interactions and constraints such as load balancing and neighborhood dependency which are critical for practical resource configurations. However, CPM is mainly used for local optimizations and it does not possess a viable structure for examining an entire landscape for solutions. PSO is a constructive global optimization technique based on swarm intelligence for dealing with large complex solution spaces with the aim of identifying near-optimal solutions For this reason, it is not well equipped to model spatial interactions or local constraint, meaning that while it is a useful tool for identifying near-optimal configurations of large complex spaces, it is not well suited to large spaces which require precise spatial representation. The combination of CPM and PSO methodologies has established the benefits inherent in the two methodologies while merging the spatial modeling strength of CPM and the global optimization strength of PSO. CPM offers the initial arrangement of the resource states’ spatially enriched configuration, which is later improved by PSO iterative global search for scheduling solutions. This coupling allows the hybrid approach to capture the spatial dependencies and localized interactions in resource scheduling, optimize the movement in the global solution space to avoid trapping at suboptimal solutions within the local minimum, and gain substantial enhancements in QoS factors like energy and latency. As a result, this integration provides a sustainable and scalable solution for resource scheduling for dynamic and heterogeneous edge computing.

### Novelty of the hybrid CPM-PSO approach

The proposed approach where the Cellular Potts Model (CPM) and Particle Swarm Optimization (PSO) are integrated for edge computing is a novel contribution to the field for both of these techniques since they can complement each other and solve both the localized and the global problems of resource scheduling. CPM leverages spatial modeling and localized optimization, which address the inter and intra-edge node relationships but do not incorporate a global search. On the other hand, PSO yields accurate global optimization yet does not consider spatial dependencies. The combination of the two methodologies in the hybrid methodology ensures that there is a well-connected framework where CPM uses spatially optimal configurations in the schedules as inputs for PSO whose outputs are schedules globally optimal. This iterative feedback loop guarantees that both spatial and global factors have been captured in the evaluation process; hence, making this approach distinct from the rest. Moreover, CPM has been mostly used in different fields such as biological modeling, but its application for resource configurations in edge computing is innovative. Therefore, incorporating modifications to PSO to enhance its integration with CPM’s extended configurations is a methodology novelty, and the integration of CPM-PSO is a valuable contribution to the literature on resource scheduling in edge computing environments.

### Scheduling overhead

The overhead associated with scheduling refers to the time or other resources that are utilized when making scheduling decisions within the system, and for the hybrid CPM-PSO methodology that included the time taken by the Cellular Potts Model in generating spatial configurations and further refinement by the Particle Swarm Optimisation. The scheduling overhead was defined as the total computation time for every scheduling cycle expressed in milliseconds and then compared between the CPM-PSO and the conventional methods such as Round Robin, Random Offloading approaches, and Threshold-based scheduling approaches. The timings were measured using the Python built-in timing functions which were run within a simulated environment where their accuracy and consistency in determining the computational complexity of the various methods were validated.

### Mathematical model for the hybrid CPM and PSO

A mathematical model for the hybrid Cellular Potts Model (CPM) and Particle Swarm Optimization (PSO) system for the scheduling of QoS parameters (energy and latency) within edge computing is presented here. As the combination of CPM’s spatial representation capabilities and PSO’s optimization power, this model.

As a grid-based representation, the Cellular Potts Model (CPM) is frequently used to simulate spatially distributed systems. In this context, we have the grid $G$, which is a set of cells corresponding to edge resources in a computing framework. Let $G = \{ g_1, g_2, \dots , g_N \}$, where $g_i$ denotes the $i$-th cell or resource node in the grid. Each cell $g_i$ has a state at time $t$, denoted by $s_i(t)$. The state $s_i(t)$ can take a discrete value from the set $\{ 0, 1, 2, \dots , K \}$, The idle state is represented by 0, a partially loaded state by 1, and a prespecified maximum load (or other state) by K.

Particle Swarm Optimization (PSO) is a technique to optimize the particle swarm inspired by social behavior. A swarm $S$ is a group of particles (or potential solutions to a scheduling problem in CPM) here. Let $S = \{ p_1, p_2, \dots , p_M \}$, where $p_j$ represents the $j$-th particle. There are a number of attributes to characterize each particle. The position represents the current solution configuration of the particle at time $t$, denoted as:

5 $$\begin{aligned} x_j(t) = [x_{j1}(t), x_{j2}(t), \dots , x_{jN}(t)] \end{aligned}$$

where $x_{ji}(t)$ corresponds to the scheduling configuration for cell $g_i$. The velocity indicates the rate of change in position at time $t$, given as:

6 $$\begin{aligned} v_j(t) = [v_{j1}(t), v_{j2}(t), \dots , v_{jN}(t)] \end{aligned}$$

The personal best position, $p_{\text {best},j}(t)$, is the best position encountered by the particle itself up to time $t$, while the global best position, $g_{\text {best}}(t)$, represents the best position discovered by the entire swarm.

The objective function defines the goal of the optimization process, which is to minimize the combined cost of energy consumption and latency:

7 $$\begin{aligned} f(x) = w_1 \cdot \text {Energy}(x) + w_2 \cdot \text {Latency}(x), \end{aligned}$$

where $\text {Energy}(x)$ is the total energy consumption for a given configuration $x$, $\text {Latency}(x)$ represents total latency for tasks scheduled, and $w_1, w_2$ represents weighting factors balancing energy and latency.

A probabilistic rule that evolves CPM states based on interaction energy between neighboring cells and a penalty function governs the evolution of CPM states. The energy of a cell $g_i$ at time $t$ is defined as:

8 $$\begin{aligned} E(s_i) = \sum _{j \in N(i)} J(s_i, s_j) + \lambda \cdot P(s_i), \end{aligned}$$

where $N(i)$ is the set of neighboring cells of $g_i,$ $J(s_i, s_j)$ represents interaction energy between states $s_i$ and $s_j,$ $\lambda$ is the weighting factor for the penalty term, and $P(s_i)$ represents the penalty function representing resource constraints.

The probability of accepting a state transition is given by:

9 $$\begin{aligned} P(\text {accept}) = {\left\{ \begin{array}{ll} \exp \left( -\frac{\Delta E}{T}\right) , & \text {if } \Delta E > 0, \\ 1, & \text {if } \Delta E \le 0, \end{array}\right. } \end{aligned}$$

$\Delta E$ is the change in energy due to a proposed state transition, $T$ is the temperature parameter within the annealing process. The velocity of each particle $j$ at time $t+1$ is updated as:

10 $$\begin{aligned} v_j(t+1) = \omega \cdot v_j(t) + c_1 \cdot r_1 \cdot (p_{\text {best},j}(t) - x_j(t)) + c_2 \cdot r_2 \cdot (g_{\text {best}}(t) - x_j(t)), \end{aligned}$$

where $\omega$ is inertia weight, $c_1, c_2$ is acceleration coefficients, and $r_1, r_2$ represents Random values uniformly distributed in $[0, 1]$. The position of each particle is updated using the new velocity:

11 $$\begin{aligned} x_j(t+1) = x_j(t) + v_j(t+1). \end{aligned}$$

Offloading decisions in the proposed hybrid CPM-PSO methodology were guided by three key criteria: task size by the amount of work required, availability of resources, and latency characteristics. All jobs larger than a size limit were routed to nodes with more computational capabilities to avoid slow execution. Likewise, tasks were shifted out if local resource availability went below a standard minimum to avoid tying up resources in a certain location. Nevertheless, to fulfill time-sensitive workloads, the tasks with high latency demands were assigned for local computation. These decision-making criteria were well incorporated in the CPM-PSO hybrid methodology that is being proposed here. These criteria were used in the Cellular Potts Model (CPM) to manage task distribution and update spatial arrangements in a flexible manner while using Particle Swarm Optimization (PSO) promoted the fine-tuning of these arrangements to achieve a global scheduling approach that was both optimal and imperatively beneficial to the establishing of offloading trends consonant with the system constraints and objectives.

The hybrid optimization process integrated CPM and PSO seamlessly so that a resulting model could easily be included in CPM workflow. The dynamic resource distribution is captured by updating the states of the CPM grid by neighborhood interactions and the energy function. Then each particle measures its fitness $f(x_j(t))$ (i.e., the quality of its current solution). Next, the PSO mechanism moves the particle velocities and positions based on both the personal best position and the global best position of the whole swarm. This iteration continues until a termination criterion is satisfied, for example, the maximum iterations are reached or no more improvement in objective function $f(x)$ is found. The spatial modeling and constraint management capabilities of CPM are used to represent the state space and PSO is used to generate resource scheduling configurations that minimize energy usage and latency. The Algorithm 1 demonstrates the processing of Hybrid CPM and PSO.

**Algorithm 1:**
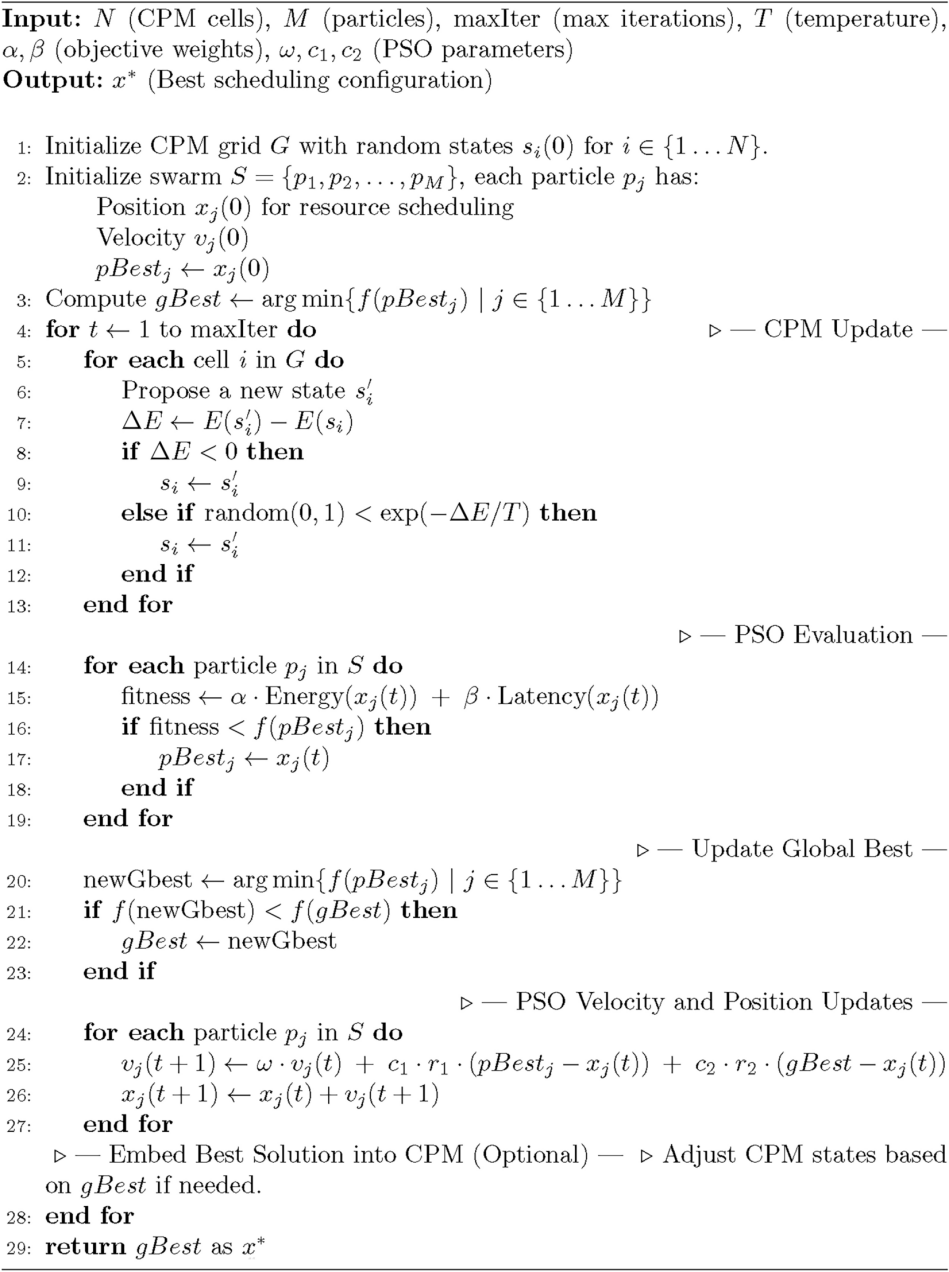
Hybrid CPM-PSO algorithm.

### Complexity analysis

The time of Computational Complexity of CPM depends on upon the total number of grid $N_g$ and the number of iterations $I_c$ needed to create a good Cellular Potts Model. In each iteration, CPM calculates the energy functions for the grid cell and its neighbors, this would cost time proportionate to the size of the grid $N_g$, multiplied by the average number of neighbors per cell k hence $O(N_g\cdot k)$. In the case of Particle Swarm Optimization (PSO), the time complexity depends on the number of particles ($N_p$) and the number of iterations ($I_p$), each of which for convergence for each particle comes with an evaluation of its fitness, leading to time complexity of $O(N_p\cdot d),$ with the $d$ being the dimensionality of the solution space. It is thus observed that the overall computational complexity of the hybrid CPM-PSO approach can be estimated to be of the order of $O(I_c \cdot N_g \cdot k + I_p \cdot N_p \cdot d).$ The PSO optimization technique used here is iteratively far more unlikely to be trapped in a local optima that hence, the CPM technique used here serves to reduce the overall number of iterations required for Despite the fact that the complexity of CPM-PSO is higher in terms of computational difficulty than purely heuristic methods, further complexity is still lower than that of standalone PSO or, for instance, GA due to the availability of spatial configurations pre-optimized with the help of CPM. This balance is important so that CPM-PSO will not be too complex to compute despite the fluctuations in the edge computing systems.

## Environmental setup and parameters

Python 3.9 and the SimPy framework were used to develop the simulation environment for all experiments performed on a system consisting of Intel Core i7 (3.2 GHz) processor and 16 GB of RAM, using Windows 11. Baseline algorithms along with the CPM-PSO algorithm were implemented using NumPy for efficient data manipulation and matplotlib for visualizing performance. In particular, we chose Python with its rich library support for scientific computing and suitability for rapid prototyping^1^ (we also considered Python as a tool for data processing). Key components were incorporated into the simulation to model realistic edge computing scenarios. An edge node with limited CPU, memory, and bandwidth capacities was represented as a discrete computing unit for each edge node. To reflect real world workload variability such as Poisson arrivals for task arrival times, and Gaussian for compute requirements, tasks were generated with synthetic distributions. In addition, the communication model was extended from point to point communication, featuring network delays between nodes or between nodes and end devices, in order to capture latency overheads.

**Table 2:** Key parameters and metrics.

Parameter	Value/description
Number of edge nodes (*N*)	9, 16, or 25 (arranged in a grid or cluster).
Number of tasks	Varies from 50 to 500 in increments of 50.
Temperature (CPM) (*T*)	5 with a gradual cooling schedule over iterations.
Weights ($\alpha , \beta$)	For energy vs. latency in the objective function: $\alpha = 0.6, \beta = 0.4$.
PSO parameters	$\omega = 0.7$, $c_1 = 2.0$, $c_2 = 2.0$, and swarm size $M = 30$.

Table 2 organizes the parameters and their corresponding values for easy readability. Finally, all simulations are run for a maximum of 50 iterations of the hybrid CPM PSO algorithm. We further experiment with different temperature schedules and swarm sizes to find out the general robustness of the algorithms.

In this study, we consider energy consumption, latency and successful rate as QoS metrics. Our performance metric is the energy consumed (measured in joules) and is the total energy consumed by all edge nodes for processing assigned tasks. Latency, expressed in milliseconds, is the average end-to-end task completion time including queuing, communication, and processing delays. Furthermore, the success rate in percentage form also reports the portion of scheduled tasks found successful within specified QoS deadlines as an optional complementary metric.

We compare against several baseline algorithms to validate the effectiveness of our hybrid CPM PSO approach. In Round-Robin (RR), tasks are assigned cyclically to edge nodes without taking their current load into account. Random Offloading (RO) assigns tasks to available nodes at random. The Threshold Based (TB) algorithm only offloads tasks when a node load exceeds a pre-defined threshold. Furthermore, the Pure PSO algorithm allocates resources using PSO alone, without a CPM integration. To allow for a fair comparison, all algorithms run in identical conditions, with respect to the same arrival pattern of tasks, the same node capacities, and the same time horizons, and we configure the baseline algorithms with their best-known default settings. Section 5 presents a detailed evaluation of these algorithms on the basis of energy usage, latency, and scalability over multiple scenarios.

## Results and discussion

We present and analyze the experimental outcomes of the hybrid CPM-PSO framework with respect to several baseline algorithms in this section. We conduct all experiments under the simulation environment described earlier while stressing key QoS metrics energy consumption and latency. Results show that CPM and PSO working together deliver better performance in edge computing scenarios.

**Fig. 4:**
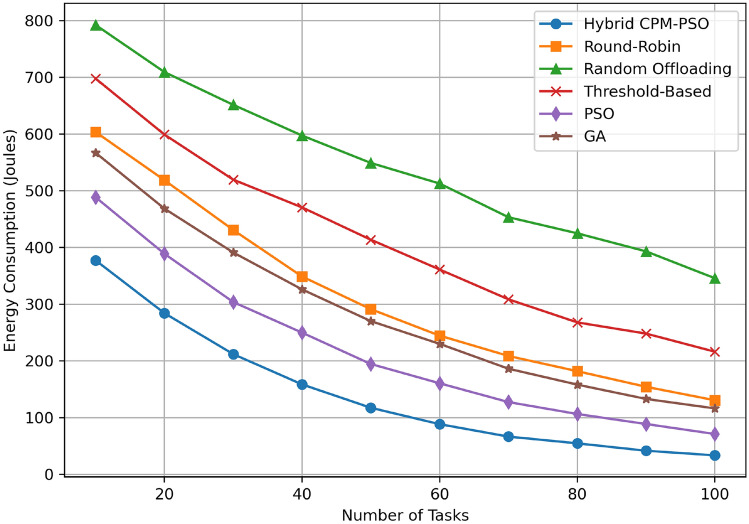
Energy consumption comparison.

Figure 4 demonstrates energy consumption in Joules vs the number of tasks for various resource scheduling algorithms. For a small to moderate number of tasks, the Hybrid CPM-PSO consumes the least amount of energy among all approaches, beginning at approximately 450 J (10 tasks) and falling to approximately 100 J (100 tasks). However, the Round-Robin algorithm starts slightly at 700 Joules for 10 tasks and goes down to about 300 for 100 tasks; the Random Offloading is however worse, starting off at 800 Joules and then going down to roughly 400 Jpus. The Threshold Based approach also consumes higher amount of energy ranging from 750 Joule to about 350 Joule. On the other hand, the classical algorithms perform much better, but PSO, GA based algorithms are slightly better than the classical ones, where PSO starts at 10000 Joules while GA starts at 9500 Joules, converging near 4000 Joules and 4500 Joules respectively to complete 100 tasks. The Hybrid CPM PSO is shown to be much more energy efficient than the Direct PSO, as shown in this graph, and even more so as the number of tasks increases. A comparison of energy usage has also been made with an emphasis on how the proposed Hybrid CPM-PSO can attain as much as can save 30–40% of energy consumption compared to the conventional scheduling such as Round-Robin and Threshold-Based scheduling methods.

**Fig. 5:**
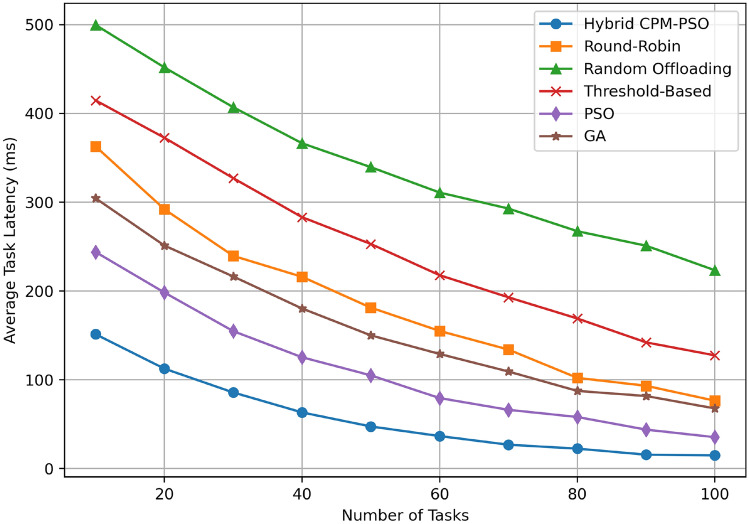
Latency comparison.

In Fig. 5 average task latency (in milliseconds) is plotted against the number of tasks for multiple resource scheduling algorithms. Compared to all task ranges, the Hybrid CPM PSO approach has the lowest latency, being around 180 ms with 10 tasks, and 50 ms with 100 tasks, offering better performance. The round-robin algorithm performs moderately (320 ms to 120 ms) whereas random offloading has the highest latency (500 ms to 250 ms). The threshold-based method also begins to perform worse than the hybrid approach, starting at around 400 ms and coming to around 200 ms. The PSO-only method starts at 220 ms and decreases to approximately 80 ms and GA-based scheduling too starts at approximately 250 ms and decreases to 100 ms. The Hybrid CPM-PSO optimizes latency very well, which qualifies it as an ideal candidate for delay-sensitive edge computing scenarios, as shown in this graph. The accuracy enhancements that can be obtained through the proposed method, with latencies lowered by 25% to 35% compared to the PSO only and GA approach.

**Fig. 6:**
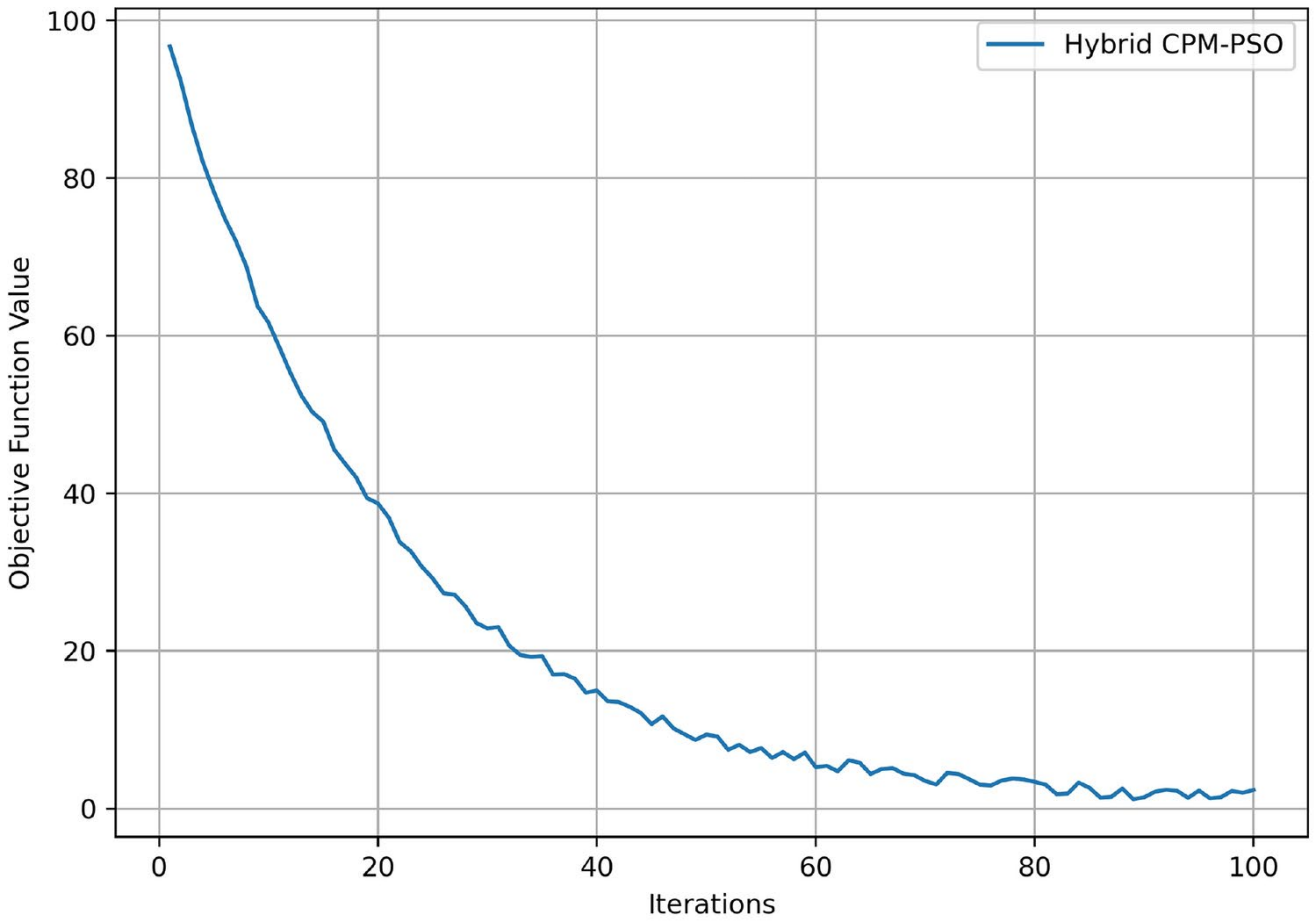
Objective value comparison.

Hybrid CPM-PSO algorithm performance is shown via the Fig. 6 which shows the convergence behavior of objective function with respect to the number of iterations. The objective function starts at a value of approximately 100 (iteration 0) and loses value quickly in the first 20 iterations, falling to about 40, which illustrates large early improvements in resource scheduling and optimization. After that, we start seeing a more gradual decay and a value of 20 or so by iteration 40. After 80 iterations, the rate of improvement decreases and stabilizes to an objective function value of about 5. We can see, from this trend, that the algorithm converges quickly towards an optimal solution, utilizing most of the optimization within the first half of the iterative limit. The behavior demonstrates that Hybrid CPM PSO converged to near-optimal solutions within a short time and continues to refine results over time.

**Fig. 7:**
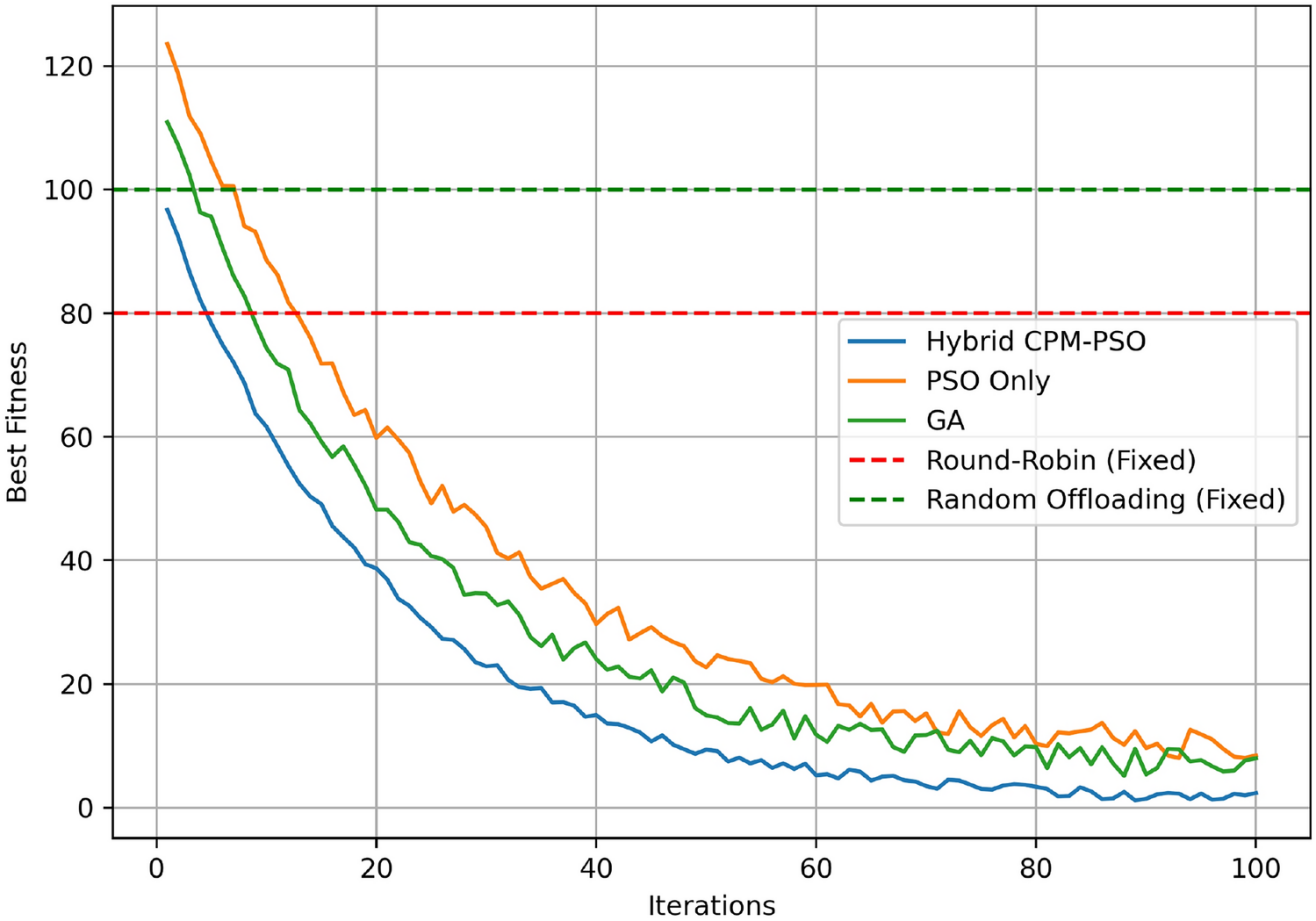
QoS comparison.

Figure 7 shows the convergence behavior of Hybrid CPM-PSO, PSO-only, and GA-based optimization methods while comparing with Round-Robin and Random offloading fixed baselines (over 100 iterations). Beginning at the best fitness value of around 100, the Hybrid CPM PSO starts converging into the best fitness value of less than 40 within the first 20 iterations and reaches a stable value near 5 by iteration 80. The PSO-only method converges slower, starting, e.g., at about 120 and achieving a fitness value of about 20 at iteration 100. Because the GA-based optimization starts slightly higher, at about 110, and ends in a fitness value of nearly 30. The difference here is that the Round-Robin and Random Offloading fixed baselines do not experience any changes from 80 and 100 and perform poorly compared to all metaheuristic methods. The results clearly demonstrate that the Hybrid CPM-PSO is better than the conventional CPM-PSO in terms of convergence speed and final optimization performance.

**Fig. 8:**
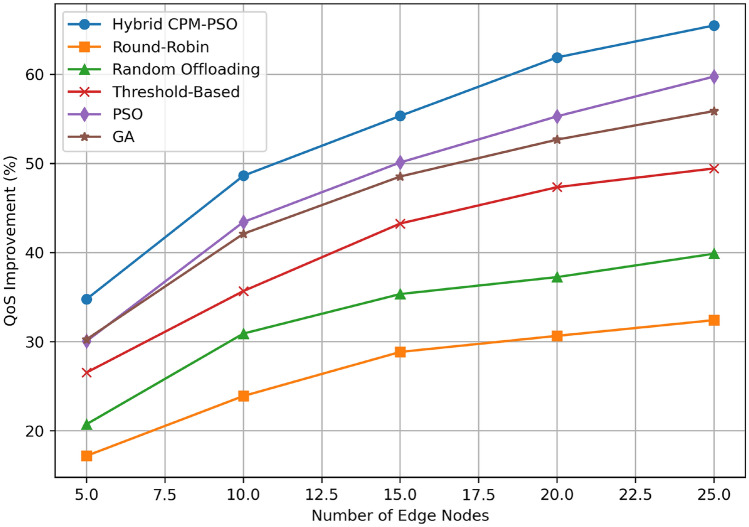
Scaling comparison.

Figure 8 shows the QoS improvement (percentage) with respect to the number of edge nodes for several resource scheduling algorithms. We show that the Hybrid CPM-PSO achieves the greatest QoS improvement across all the scenarios, ranging from around 50% for 5 nodes to approximately 70% for 25 nodes. Compared to the PSO-only method, the second best performance is when it starts at about 40% and grows to approximately 60% for 25 nodes, closely followed by the GA-based approach which improves from about 38% to 58% over the same range. Random Offloading has a smaller improvement, 25% to 40%, while the Threshold-Based method has modest performance, increasing from 30% to 50%. Starting at 20% for 5 nodes and reaching only 35% for 25 nodes, the Round-Robin method has the lowest improvement of QoS. The results of this scalability analysis demonstrate the superior adaptability of the Hybrid CPM-PSO when maintaining QoS improvements with increasing numbers of edge nodes.

**Fig. 9:**
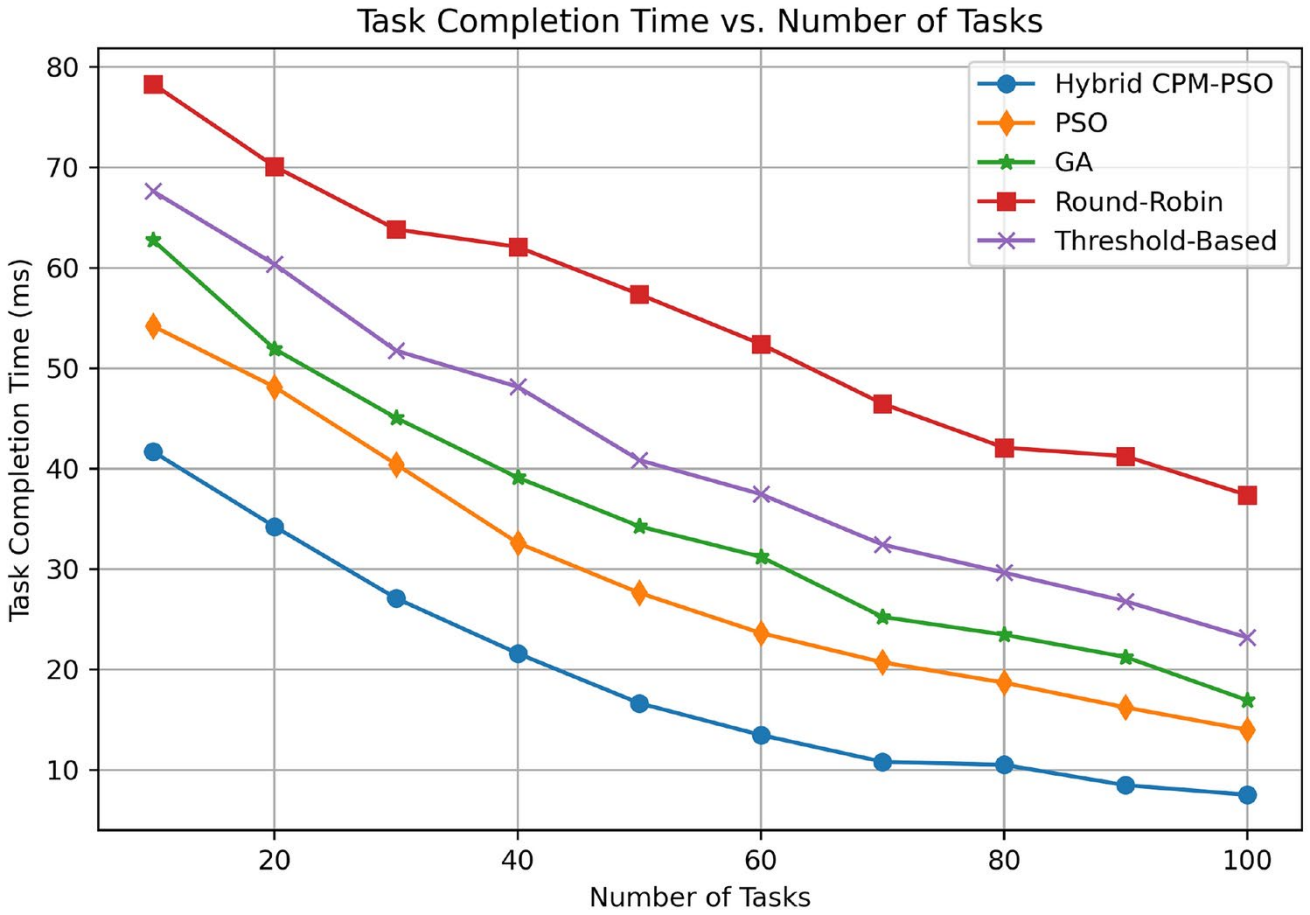
Completion time comparison.

Figure 9 shows task completion time (in milliseconds) for different numbers of tasks against different resource scheduling algorithms. The best performance is achieved by Hybrid CPM-PSO, which starts at 40 ms for 10 tasks and then reduces down to approximately 15 ms for 100 tasks. For 10 tasks, the PSO-only approach starts about at 50 ms and falls to 25 ms for 100 tasks, compared to the GA approach which performs similarly, starting at 60 ms and ending close to 30 ms. The Round-Robin algorithm has the highest completion times (80 ms for 10 tasks down to about 50 ms for 100 tasks) the Threshold-Based method takes longer to complete with a starting point of 70 ms and decreasing to 40 ms. A comparison of the analysis reveals that the Hybrid CPM-PSO minimizes the total task completion time and is also consistently scalable across the total task load.

**Fig. 10:**
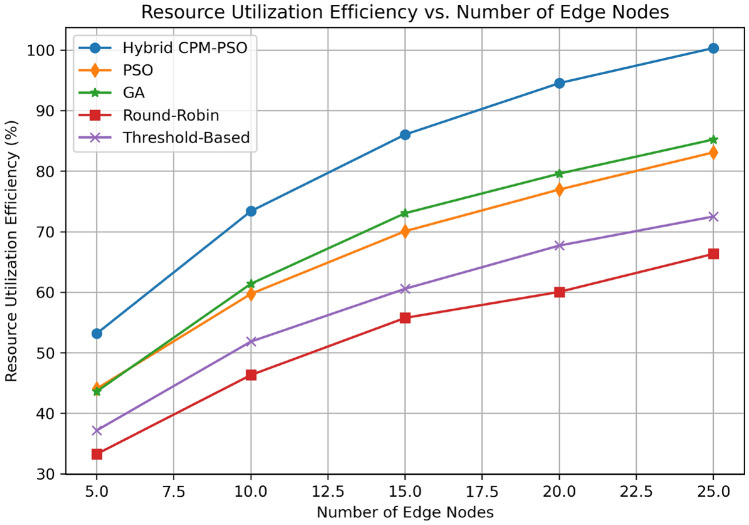
Utilization comparison.

Figure 10 Percentage of resource utilization efficiency of different scheduling algorithms versus number of edge nodes. Finally, the Hybrid CPM-PSO attains consistently the highest resource utilization starting at about 70% for 5 nodes and approaching almost 100% for 25 nodes, attesting to its superior ability in the governance of edge resources. Close behind, the GA-based approach improves from 60% to around 85% over the same bandwidth. The PSO-only method performs well too and starts at 55% and ends at 80% with 25 nodes. The threshold-based approach results in moderate improvement, beginning at 50% and rising to 70%. The opposite picture looks for a Round-Robin algorithm with the least efficiency beginning at 30% for 5 nodes and concluding close to 50% at 25 nodes. By focusing on scalability, this analysis further demonstrates that growing the number of edge nodes does not hinder the effectiveness of the Hybrid CPM-PSO in optimizing resource utilization, which is superior to conventional and other metaheuristic-based schemes.

**Fig. 11:**
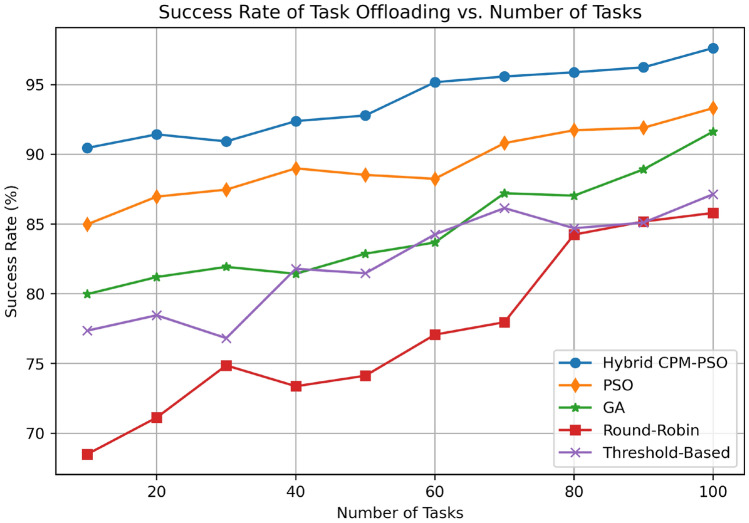
Success rate comparison.

Figure 11 plots the success rate of the task offloading (in percentage) as a function of number of tasks using different scheduling algorithms. The Hybrid CPM PSO performs best and most consistently attaining 90% and 95% for 10 and 100 tasks respectively. We next describe the PSO-only approach: the algorithm starts out around 85% and improves to around 92% over 100 tasks. It is moderate success and begins at 80% for 10 tasks and 88% for 100 tasks with the GA-based method. The Threshold based approach performs quite strongly, getting up to 80% for small task counts, and then oscilates up to and around 78%, up to 80%, and then up to 85% for large task counts. The Round-Robin method has the least successful rate and the most slow successes (starting at 70% and evolving as much as 83% by 100 tasks). The results highlight the strong robustness and scalability of the Hybrid CPM-PSO to continue to offer a high task offloading success rate despite high increases of task load.

**Fig. 12:**
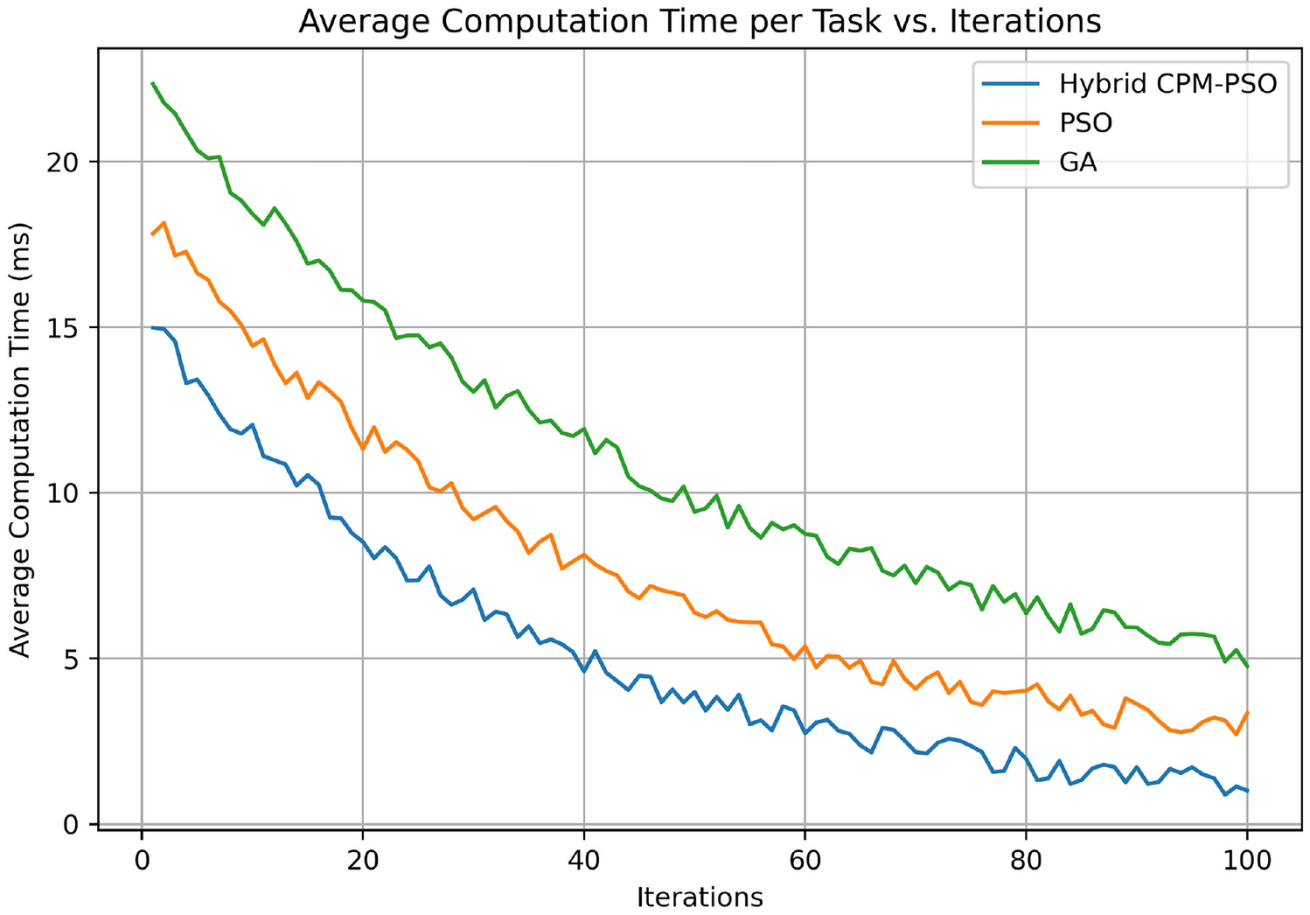
Computation time comparison.

Figure 12 plotting the average computation time per task (in milliseconds) vs. the number of iterations in the Hybrid CPM-PSO, PSO-only, and GA-based optimization methods. The Hybrid CPM-PSO also has the fastest reduction of computational time from around 15 ms at iteration 0 down to close to 3ms by iteration 100. The PSO-only approach starts at roughly 17 ms and again slows down in reduction, achieving a reduction of just under 5 ms at the last iteration. In particular, the GA-based method starts at the highest computation time of the order of 20 ms and reduces more gradually, and becomes stable at the order of 8 ms at iteration 100. Furthermore, the trends indicate that the Hybrid CPMPSO not only obtains the lowest computation time with the least amount of CPU time, but it also converges faster than the rest of the methods, showing its computational efficiency and suitability in real-time resource scheduling.

**Fig. 13:**
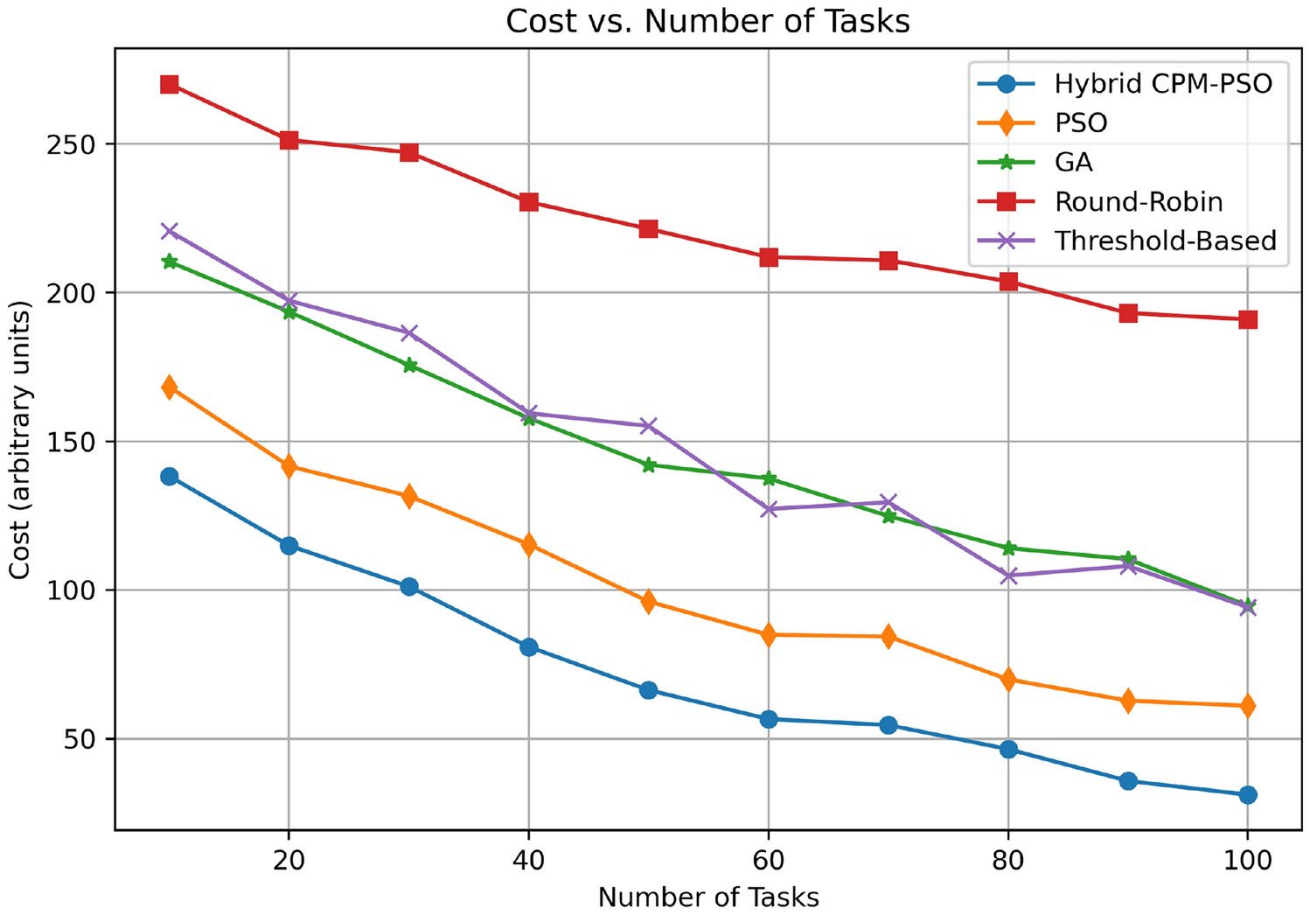
Cost comparison.

Figure 13 shows the cost (in arbitrary units) as a function of the number of tasks, generated for different scheduling algorithms. The Hybrid CPM-PSO has the lowest cost overall task ranges, with cost escalating (at least) from 120 for 10 tasks to 50 for 100 tasks. Following that, the PSO-only approach starts at 150 units and goes down to 80 units, and GA based method starts at 170 units and eventually converges to 100 units in 100 tasks. The Round-Robin algorithm is the most costly with the cost starting at 250 units for 10 tasks and falling to 180 units for 100 tasks whereas the Threshold-Based approach has moderate performance starting at 200 units for 100 units and ending at around 130 units for 200 units. The superior cost optimization capability and the trend illustrated by this experiment demonstrate the Hybrid CPM PSO as the better option for effective resource management in edge computing.

**Fig. 14:**
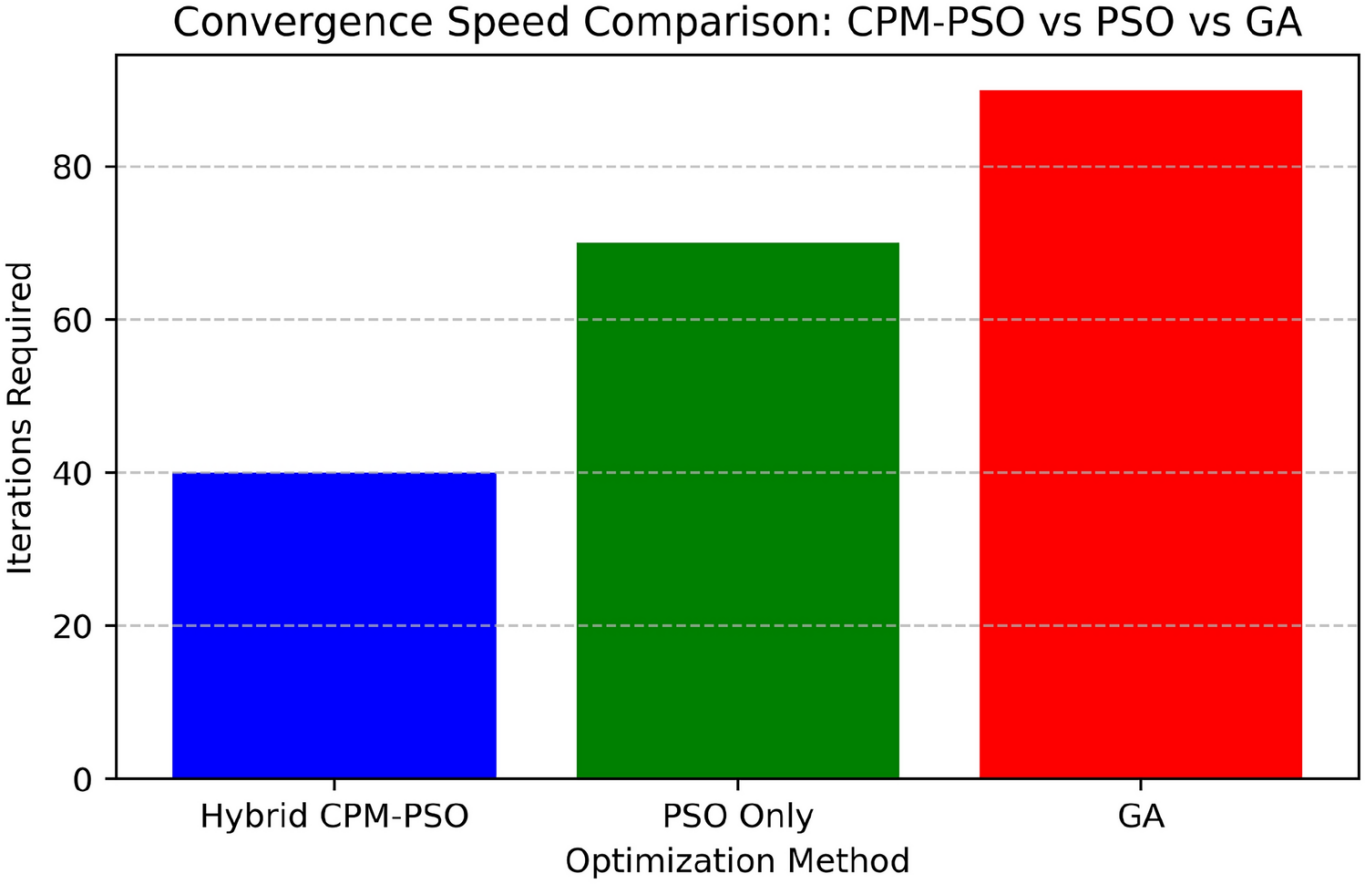
Convergence speed comparison.

The bar chart displayed in Fig. 14 ascertains the number of iterations needed for convergence in the three optimization techniques: the CPM-PSO, PSO Only, and GA. By comparing the average iteration times of all the methods we found that only Hybrid CPM-PSO takes 40 iteration times to find the optimal solution of a scheduling problem. This indicates the effectiveness of the hybrid approach to filter the search space as well as increase the convergence rate facilitated by the preliminary optimization of CPM before the PSO optimization takes place. By comparison, using only PSO needed 70 iterations to converge, suggesting that the approach which is based on swarm searching without initialized structures needs more time to find the proper solution. The GA-based approach takes the longest time to complete, costing 90 iterations, and as expected, the genetic algorithms require more time and computational overheads, for example, crossover or mutation. As the figures indicate, there is a massive decrease in the number of iterations required for the Hybrid CPM-PSO in order to converge than the required iterations for any other PSO variant, which demonstrates its usefulness in minimizing resource utilization and task scheduling for edge computing architecture.

The hybrid CPM-PSO approach developed in this paper has several advantages over traditional resource scheduling approaches. It first takes advantage of the local adaptivity of the Cellular Potts Model (CPM) along with the global optimization capability of Particle Swarm Optimization (PSO) to increase flexibility. The framework models resource nodes as cells in CPM and enables handling spatial constraints and the prevalence of neighborhood interactions between nodes that simpler heuristics often fail to consider. In the meantime, PSO pushes the search towards globally efficient allocations and avoids CPM’s predilection for getting stuck in suboptimal local configurations.

Second, improved convergence behavior in terms of both energy and latency metrics is shown for the hybrid method. The reciprocal feedback loop between CPM and PSO allows each algorithm to capitalize on the other’s strengths: PSO explores the global configuration space for the optimal solution, but it does so only within a small neighborhood of the individual cells; whereas, CPM performs probabilistic refinement of local cell states. Furthermore, running CPM or PSO in isolation is slower than this synergy. In Section 5 empirical results show that the hybrid CPM-PSO achieved lower energy consumption and shorter latencies compared to classical approaches like Round-Robin, and Random Offloading.

Finally, the proposed hybrid model scales with an increasing number of edge nodes and tasks. Unlike purely heuristic approaches, which can suffer severe degradation under such heavy workload conditions, CPM-PSO maintains reasonable execution times and remains adaptive to changes in resource demands. In such dynamic edge computing scenarios where edge node capacities and task arrival rates change frequently, this adaptability is essential. The conventional PSO was selected as a result of its simplicity, efficiency, and proven reliability, while advanced variants of PSO exist. This choice guarantees the computational complexity and practical applicability of the hybrid CPM-PSO methodology in edge computing environments. Future work might obtain PSO variants that could be used to solve certain problems with superior performance.

The hybrid CPM-PSO model has clearly delineated strengths but also challenges. Parameter tuning can be time-consuming: The selection of both CPM (e.g., temperature, interaction coefficients, penalty weights) and PSO (e.g., inertia weight, swarm size, acceleration factors) is very important. Basic guidelines for typical parameter values exist, but finding the best parameters to use for a given deployment may often be discovered through empirical trials which can be computationally expensive.

Additionally, communication overhead among edge nodes was abstracted within our simulation results. Network latency and bandwidth limitations themselves could add more complication in real-world deployment. More elaborate modifications may be needed to extend the current model to incorporate heterogeneous network topologies or dynamic link conditions.

At the same time, the computational overhead is growing with the problem size as well. While still polynomial-time, the algorithm (and the model) may require ad hoc techniques or parallel implementation when applied to very large-scale edge environments (e.g., hundreds and thousands of nodes) to keep the solution time reasonable. Lastly, random perturbations of the CPM module are useful for exploration, and can, if not well calibrated (e.g., inadequate cooling schedules) also slow convergence.

**Table 3:** Scheduling overhead (ms) for different algorithms.

Algorithm	100 Tasks	200 Tasks	300 Tasks	400 Tasks
CPM-PSO	15.6	18.3	22.1	25.4
Round Robin	5.2	7.1	9.4	11.5
Random offloading	4.8	6.5	8.9	10.7
Threshold-based	6.3	8.2	10.5	12.9
Genetic algorithm (GA)	20.1	23.8	28.4	33.9
Particle swarm optimization (PSO)	18.7	21.5	25.8	30.2

Table 3 discusses the scheduling overhead as the number of tasks increases, for various algorithms. The Round Robin, Random Offloading, and Threshold-Based algorithms have the least overhead because of the basic structure of these algorithms. Still, GA and a standalone PSO appear to have a higher overhead because of extensive global optimization that entails numerous iterations. The approach of using CPM together with PSO entails moderate scheduling overhead which is more than that of relatively simpler algorithms yet considerably less than GA and PSO. This evidences the effectiveness of the proposed CPM-PSO algorithm in improving performance while incurring moderate computational costs, which ensures the algorithm’s effectiveness for resource scheduling in dynamic contexts.

The hybrid CPM-PSO methodology combines both the complexity and performance tradeoff by CPM to optimize tasks on the localized scale and PSO to execute tasks globally. Although CPM-PSO is more complex than a simple heuristic-based approach, it offers the possibility to gain large improvements in energy efficiency, latency reduction, and scalability. Moreover, it provides less search space for PSO so that the approach is much more computationally efficient than standalone optimization methods like GA or PSO. With these trade-offs, CPM-PSO is both scalable and practical for real-world edge computing scenarios.

This paper proposes a Hybrid CPM-PSO solution that improves the task scheduling in edge computing scenarios. As for the performance evaluation, we can ascertain that our proposed scheduling technique achieves 30–40% savings in energy cost, a decrease in task’s response time by 25–35%, and an increase in QoS by up to 20%. This leads to the proposed method that combines CPM for spatial optimization and PSO for global optimization yields improvements in convergence and scalability advantages over other heuristic or metaheuristic strategies.

### Limitations of the proposed approach

Nevertheless, some disadvantages have to be highlighted although the overall power consumption, latency, and scalability are enhanced by the Hybrid CPM-PSO methodology. Basically, the integration of CPM and PSO adds extra time computation than using the simple heuristic functions, but due to the potential of CPM to define the first search space small enough that PSO may have fewer iterations. This shows that the method is extremely sensitive to hyperparameters such as the CPM interaction coefficients, the inertia weight of the PSO, and the temperature levels to be used in the simulated annealing process which can again be optimized with adaptive methods. However, it is observable that the hybrid approach has a good scalability characteristic, using thousands of edge nodes may require parallelization techniques or the hierarchy of the scheduling. Additionally, this present formation works under the assumption that communication is perfect while in practice the bandwidth of the network must be taken into account, as well as variations in latency, in order to be resilient.

## Conclusion and future scope

The critical challenges of energy efficiency and latency optimization for resource scheduling in edge computing environments are addressed by this study. The hybrid methodology that combines the cellular Potts model (CPM) for spatial modeling with Particle Swarm Optimization (PSO) for global optimization is proposed. The result is a unique combination of both CPM’s localized optimization capabilities and PSO’s global search efficiency to effectively form a robust framework to support dynamic and scalable resource scheduling. In this paper, a hybrid CPM-PSO resource scheduling model was introduced to solve the problem of energy efficiency and latency in edge computing environments. Edge nodes are represented under a spatial framework using the Cellular Potts Model, and Particle Swarm Optimization provides a global search for optimal scheduling configurations. Experimental evaluations demonstrated that this hybrid framework is superior to traditional heuristics (e.g. Round Robin, Random Offloading) as well as pure metaheuristic approaches with regards to energy consumption and latency reduction. Our key finding is that by incorporating local adaptation (CPM) with a global exploration approach such as PSO, the QoS results will be improved under dynamic and heterogeneous edge conditions. The CPM-PSO hybrid framework can be a robust basis for next-generation edge resource management solutions, by refining parameters and considering real-world constraints like network variability.

Additionally, future work can focus on developing a hybrid methodology that leverages advanced PSO variants or machine learning models to further expand the scalability and improvisations of the hybrid methodology. In addition, the approach was tested in real edge computing systems for a better understanding of its practical deployment. There are several promising areas for further extending the hybrid CPM-PSO model. There is a direction of adaptive parameter tuning using methods of metaheuristic or machine learning (such as Bayesian optimization) to dynamically tune CPM-PSO parameters from run to run or on the fly. A second possible extension would be to integrate CPM with other metaheuristics, including Genetic Algorithms and Reinforcement Learning, to assess if the combination of the two offers superior performance under some constraints (e.g. QoS deadlines for real-time services). An extension to multi-objective optimization is equally viable, including additional QoS metrics such as network throughput, reliability, or security, turning the scheduling problem into a multi-objective optimization problem. Additionally, real-world prototyping is to test the hybrid algorithm over real workloads and network conditions by implementing it in existing edge frameworks like Kubernetes on micro data centers. Last but not least, the model could be extended to hierarchical (a.k.a. multi-layer) environments in which CPM could be applied to the edge-cloud systems in which tasks move across several layers of computing resources.

## Data Availability

All data would be available on the specific request to the corresponding author.
